# Comparative Genomics, Transcriptome, and Prokaryotic Expression Analysis of *alk*B1_1 in *Acinetobacter vivianii* KJ-1: Revealing the Mechanism of Petroleum Hydrocarbon Degradation

**DOI:** 10.3390/ijms26094083

**Published:** 2025-04-25

**Authors:** Qiannan Cui, Yali Zhang, Jie Wang, Jianing Wang, Qingqing Zhao, Fanyong Song, Leilei Wang, Wen Zhang, Yujie Huang

**Affiliations:** Shandong Provincial Key Laboratory of Applied Microbiology, Ecology Institute, Qilu University of Technology (Shandong Academy of Sciences), Jinan 250103, China; cui17344805862@163.com (Q.C.); ya_zyl@163.com (Y.Z.); wxyh_888@163.com (J.W.); wangjn@sdas.org (J.W.); qingqingzhao@qlu.edu.cn (Q.Z.); aiyoutianyi01@163.com (F.S.); heat_33wll@163.com (L.W.); zw-sunshine@163.com (W.Z.)

**Keywords:** *Acinetobacter vivianii*, comparative genomics, transcriptome analysis, prokaryotic expression, alkane degradation

## Abstract

The present study aimed to comprehensively dissect the petroleum hydrocarbon degradation mechanism of *Acinetobacter vivianii* KJ-1. The isolated and identified strain was able to proliferate using diesel as the sole carbonaceous substrate. Via comparative genomics, an in-depth analysis was performed to elucidate the genome similarities and disparities between this strain and related strains, thereby uncovering a core genome as well as genes with uncharacterized functions. Transcriptome analysis, carried out under different substrate conditions (C16, diesel, sodium acetate) manifested distinct gene expression modalities. A multitude of genes associated with alkane metabolism were differentially expressed, among which *alk*B1_1 and *alk*B1_2 was conspicuously upregulated. Prokaryotic expression of *alk*B1_1 was implemented, and the enzyme activity of the recombinant protein peaked at a pH level of approximately 7.0 and within a temperature range of 30 to 40 °C. The recombinant strain was shown to possess the ability to degrade n-hexadecane. Collectively, this research not only augments the understanding of the degradation mechanism of *A. vivianii* KJ-1 but also provides a fundamental basis for developing bioremediation strategies targeting petroleum hydrocarbon-contaminated environments.

## 1. Introduction

Petroleum hydrocarbons, as delineated by Hoang [1], serve as prominent constituents within the realm of persistent organic pollutants. In the event of leakage into adjacent terrestrial areas, the oil-bearing substances that permeate the soil matrix possess the propensity to disrupt the soil structure, leading to a reduction in soil porosity and a consequent attenuation of permeability, as well as inducing alterations in the soil’s physical characteristics [2]. Concurrently, the reactive moieties present within the oil-bearing substances can engage in chemical interactions with inorganic nitrogen and phosphorus species extant in the soil. This chemical interaction, in turn, gives rise to a decline in the soil’s organic matter content. Eventually, such perturbations culminate in the disturbance of the microbial ecological niche, thereby impinging upon the survival and viability of microorganisms and curtailing overall soil biological activity [3,4]. These pollutants harbor highly toxic components and manifest a “triple effect”, namely, teratogenicity, carcinogenicity, and mutagenicity. As a corollary, they impose a certain degree of detriment to health and well-being of both human and animal populations [2,5].

Bioremediation technology pertaining to petroleum hydrocarbon pollution provides several advantages, namely, straightforward operational procedures, cost-effectiveness, and the absence of secondary pollution [6]. These attributes confer upon it a preeminent status as one of the most prevalent and prospective remediation technologies in the contemporary context [7,8]. The employment of bioremediation technology for the remediation of sites contaminated with petroleum hydrocarbons is predicated on the fact that a multitude of components within petroleum hydrocarbons can be metabolized by microorganisms [6]. In the natural environment, upon contamination with petroleum hydrocarbons and other organic pollutants, the biological community undergoes alterations. This gives rise to a substantial augmentation in the population of non-dominant microorganisms, especially bacteria, under the impetus of the external environment [9]. These bacteria possess the capacity to metabolize and proliferate by utilizing petroleum hydrocarbons as substrates, thereby fulfilling a crucial function in the degradation of petroleum hydrocarbon [10,11]. Currently, more than 90 genera and over 200 species of bacteria endowed with the ability to degrade petroleum hydrocarbons have been reported, including *Pseudomonas* spp., *Bacillus* spp., *Alcanivorax* spp., and *Acinetobacter* spp., among others [12,13,14].

*Acinetobacter* constitutes a genus of bacteria that plays a significant and pivotal role in the degradation of petroleum hydrocarbons [15,16]. A multitude of studies have been carried out to isolate and characterize *Acinetobacter* strains with the capacity to degrade various components of petroleum, including n-alkanes and crude oil. In addition, genes, enzymes, and associated metabolic pathways implicated in petroleum hydrocarbon degradation have also been the focus of in-depth analysis. For example, *Acinetobacter mesopotamicus* sp. nov. has been shown to proficiently degrade different crude oils by means of three alkane hydroxylases (AlkMa, AlkMb, and AlmA) for specific n-alkane oxidations [17]. *Acinetobacter oleivorans* DR1 is able to adhere to and proliferate on diesel oil, and its metabolic and stress responses during the degradation of long-chain alkanes have been investigated [18]. Furthermore, *Acinetobacter calcoaceticus* CA16 can flourish in minimal media supplemented with diesel as the sole carbon source, and degrade 82% to 92% of aliphatic alkane hydrocarbons (ranging from C12 to C18) within 28 days [19].

In the exploration of the role of *Acinetobacter* in hydrocarbon degradation, transcriptomics and comparative genomics assume paramount significance in deciphering the degradation mechanisms of petroleum hydrocarbons by *Acinetobacter* spp. [20]. Comparative genomics facilitates the identification of genetic elements and pathways that are distinctive to different strains of *Acinetobacter* and are implicated in petroleum hydrocarbon degradation [21,22]. Through the comparison of genomes of various strains, researchers can identify genes and operons that are either conserved or display differential expression, thereby affording valuable insights into the molecular substrates of hydrocarbon degradation [23]. Transcriptomics, in contrast, allows for the analysis of gene expression patterns in response to exposure to petroleum hydrocarbons. This methodology facilitates the identification of genes that are either upregulated or downregulated during the degradation process, providing a temporal snapshot of the active metabolic pathways at a specific time [24,25]. Collectively, comparative genomics and transcriptomics represent a powerful instrument for apprehending the complex interactions between *Acinetobacter* and petroleum hydrocarbons, as well as for formulating strategies for bioremediation.

During the incipient phase of our research, a strain designated as KJ-1, which manifested the capacity to degrade petroleum hydrocarbons, was successfully procured through a screening protocol. This strain was proficient in rapid proliferation within an inorganic salt medium, with diesel functioning as the sole carbonaceous substrate, and was subsequently taxonomically identified as *A. vivianii* [11]. Via whole-genome analysis, a plurality of genes associated with petroleum hydrocarbon degradation were discerned, among which were two alkane-1-monooxygenase encoding genes, namely *alk*B1_1 and *alk*B1_2, renowned for their instrumental role in alkane degradation. Grounded on the outcomes of the preliminary research, in the present study, we amalgamated comparative genomics and transcription analysis to attain a more profound comprehension of *Acinetobacter vivianii.* Additionally, we endeavored to explore the prokaryotic expression of the *alk*B gene. The overarching objective is to erect a theoretical scaffolding for further inquiries into the molecular mechanisms underlying the strain’s metabolism of petroleum hydrocarbons.

## 2. Results

### 2.1. Degradation of Various Alkanes with A. vivianii KJ-1

**Figure 1:**
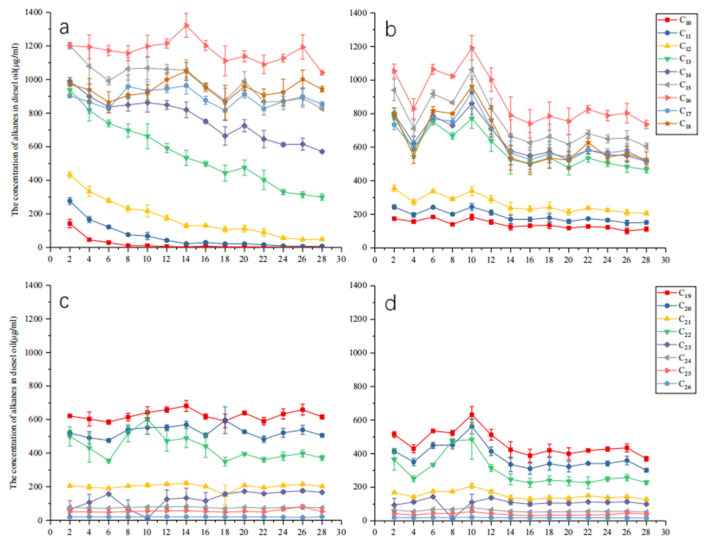
The variation in alkane concentration over time in untreated and treated samples with KJ-1. (**a**,**c**) Untreated; (**b**,**d**) Treated with KJ-1.

In the MSM with diesel as the sole carbon source, the alkane levels in treated and untreated samples were determined. As shown in Figure 1, certain alkane components in diesel changed after treatment. Figure 1a,c show the alkane abundances in the control group. Diesel mainly consists of C10–C26 alkanes, with higher C13–C20 alkanes. Short-chain C10–C12 alkanes evaporated over time. After treatment with strain KJ-1 (Figure 1b), their concentrations first decreased in the initial 4 days and then increased on the 6th day, likely due to long-chain alkane degradation. Compared to the control group without strain KJ-1, the levels of long-chain alkanes (C14–C26) were reduced (Figure 1b,d). To thoroughly explore the degradation mechanism of hydrocarbon-degrading bacterium *A. vivianii*, its genome and related degradation proteins with those of other petroleum hydrocarbon-degrading *Acinetobacter* species were compared.

### 2.2. Genomic Comparison

**Figure 2:**
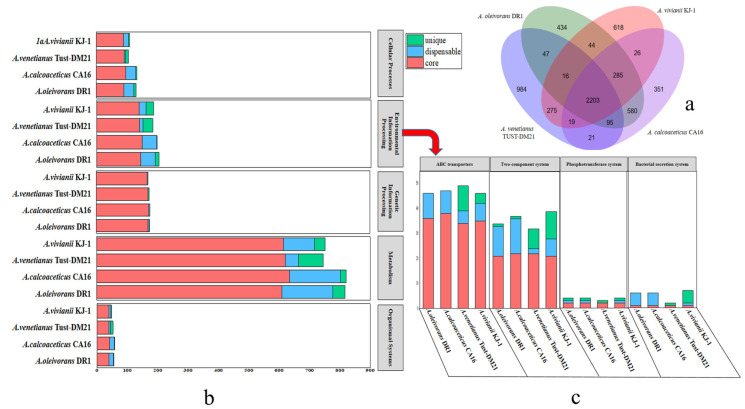
Genomic comparative analysis of *Acinetobacter* spp. (**a**) Venn diagram of genomic analysis of *Acinetobacter* spp., (**b**) Gene distribution based on KEGG classification, (**c**) Distribution of genes related to environmental information processing in the KEGG classification system.

The genomic traits of the four strains listed in Table 1 exhibited both similarities and differences. Specifically, the fluctuation ranges of the genome size and the number of genes encoding proteins were less than 5.60% and 6.33%, respectively. A comparative analysis regarding the homology of the predicted protein-coding genes revealed significant gene overlap among the four genomes. The core genome is composed of 2203 genes, accounting for 57% to 61% of the total number of genes in these four genomes (as shown in Figure 2a). There are the largest number of orthologous genes between *A. vivianii* KJ-1 and *A. venetianus* TUST-DM21. According to the classification of KEGG annotation, the genes of these four strains are mainly related to metabolism, genetic information, and environmental information processes (Figure 2b). Compared with strains DR1 and CA16, strain KJ-1 has more unique genes in the classification of environmental information processes, especially in the two-component system (Figure 2c).

**Figure 3:**
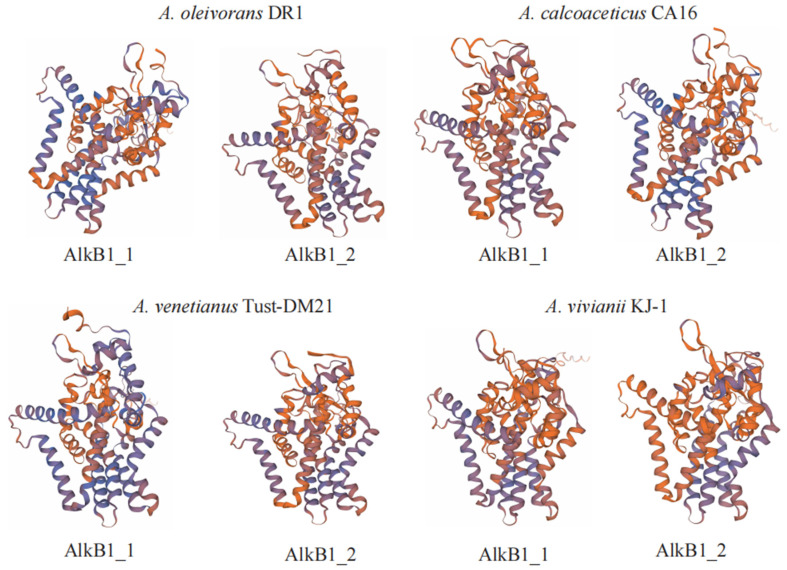
Three-dimensional structural diagrams of AlkB proteins derived from various *Acinetobacter* species.

AlkB is a key enzyme in metabolism [26]. In *A. vivianii* KJ-1’s genome, two genes encoding alkane 1-monooxygenase were found. A comparison of its AlkB proteins showed that they all have the conserved HYG-motif (N[Y/F][I/L]EHYG) and three Hist-box motifs (Hist-A: HEL[S/G]HK; Hist-B: EH[P/N][Y/R]GHH; Hist-C: LQRHSDHHA) [11]. Using AlkB1 in *A. baylyi* ADP1 tertiary structure as a template, SWISS-MODEL (https://swissmodel.expasy.org/ accessed on 7 November 2022) simulated the tertiary structure of eight AlkBs from four *Acinetobacter* strains (Figure 3). All eight AlkB proteins had six transmembrane helical regions, consistent with previous reports [27]. Analyzing conserved loci and transmembrane regions confirmed that the HYG and three histidine box structures were in the cell’s hydrophilic domains. Six transmembrane helical regions and the HYG histidine box are features of alkane monooxygenase [27]. AlkBs not only start the catabolic process via demethylation but also increase pollutant bioavailability [28]. To verify AlkB’s role in *A. vivianii’s* alkane degradation, besides genomic comparisons with other *Acinetobacter* species, transcriptomics studies were done on *A. vivianii* KJ-1 with different carbon sources. Also, the gene encoding AlkB was expressed in prokaryotes. These efforts aim to better understand how AlkB works in this bacterium’s degradation process.

### 2.3. General Features of Transcriptome in Different Carbon Sources

Strain KJ-1 was cultivated in inorganic salt media using n-hexadecane (C_16_), diesel, and sodium acetate as substrates, respectively, for a certain time. Subsequently, the transcriptome was isolated via RNA-seq technology. The three groups of samples yielded 7,084,306 to 14,419,842 clean reads, covering 78.11% to 98.26% of the genome (as detailed in Table S2).

Genes with transcripts per million (TPM) values of 1 or more were considered expressed. Gene expression of strain KJ-1 induced by different substrates was analyzed. Based on the statistical results, it was found that strain KJ-1 harbored a total of 3716 genes. When sodium acetate (Control_SA) was utilized as the sole carbon source for cultivation, 3626 genes (accounting for 97.58% of the total genes) were expressed within the transcriptome. In the case where n-hexadecane (Sample_C_16_) or diesel oil (Sample_Dio) served as the sole carbon source, 3669 and 3677 genes were transcribed, respectively. Moreover, 57 novel genes were expressed in both Sample_Dio and Sample_C_16_ transcriptomes.

**Figure 4:**
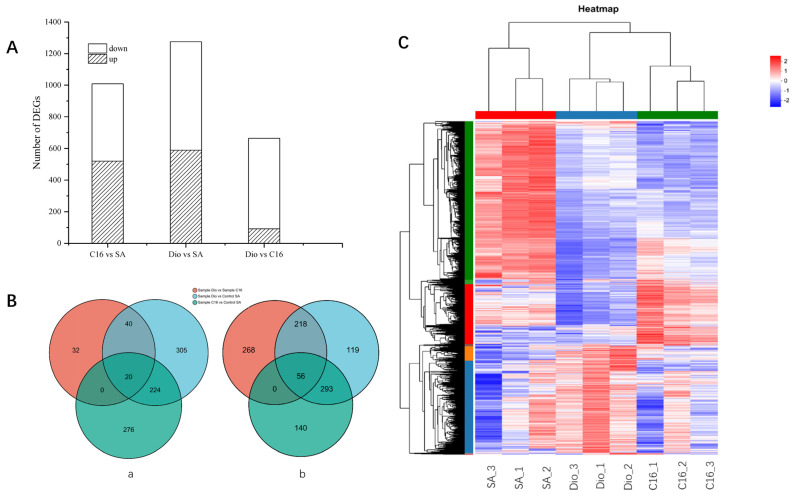
Overall transcriptional variations of KJ-1 in growth conditions with n-hexadecane (C_16_), diesel oil, and sodium acetate. (**A**) The quantity of DEGs under two different hydrocarbons in comparison to sodium acetate was the control. In this visualization, the white bars represent the downregulated genes, while the grey bars denote the upregulated ones. (**B**) Venn diagram illustrating the distribution of the upregulated and downregulated DEGs when the strain KJ-1 is cultured with C_16_ and Dio in comparison to sodium acetate. Here, ‘a’ refers to the upregulated DEGs under diverse conditions, and ‘b’ indicates the downregulated DEGs under different circumstances. (**C**) Heat map of the transcription profiles of genes in strain KJ-1 under C_16_, Dio, and sodium acetate. The intensity of the color reflects the magnitude of gene expression within the sample, as per the color scale provided on the upper right.

Differentially expressed genes (DEGs) were screened using sodium acetate (SA) as a control for diesel and n-hexadecane (Dio vs. SA, C16 vs. SA), and also between diesel and n-hexadecane (Dio vs. C16) in *A. vivianii* KJ-1. Based on the analysis scheme (Dio vs. SA, C_16_ vs. SA, and Dio vs. C_16_) and the criteria (|log_2_FC| ≥ 1), a total of 1009 and 1275 DEGs were identified. Comparing the C_16_/diesel with SA, 520/489 and 589/686 DEGs were found to be up/down regulated (as shown in Figure 4A). Only 244/349 DEGs were co-up/down regulated when strain KJ-1 was cultured with C_16_ and diesel oil as substrates compared to sodium acetate (depicted in Figure 4B). When these two samples were compared (Dio vs. C_16_), there were 664 DEFs (92 up, 572 down) (Figure 4A,B). In the culture medium with diesel as the substrate, more transcripts of *A. vivianii* KJ-1 are regulated. During the first three days, *A. vivianii* KJ-1 preferentially utilizes short-chain alkanes. At this stage, genes related to alkane degradation in the transcriptome are expressed, resulting in genes related to long-chain alkane degradation being downregulated when compared to n-hexadecane. Cluster analysis demonstrated that the genes were clustered according to their respective growth conditions (Figure 4C).

### 2.4. Analysis of Upregulated DEGs

**Figure 5:**
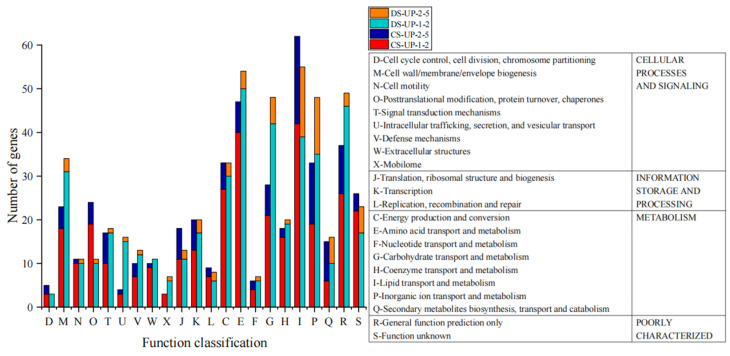
COG classification of upregulated DEGs in the different groups. CS-UP-1-2 means the upregulated DEG number with 1 ≤ log_2_(FC) < 2 in the C_16_ vs. SA group, CS-UP-2-5 means the upregulated DEG number with 2 ≤ log_2_(FC) < 5 in the C_16_ vs. SA group, DS-UP-1-2 means the upregulated DEG number with 1 ≤ log_2_(FC) < 2 in the Dio vs. SA group, and CS-UP-2-5 means the upregulated DEG number with 2 ≤ log_2_(FC) < 5 in the Dio vs. SA group.

The criteria for genes to be regarded as significantly differentially expressed and upregulated are as follows: log_2_(FC) ≥ 1 and p_adjust < 0.05. In the C_16_ vs. SA group, there are three genes with log_2_(FC) ≥ 5. Two are hypothetical proteins, and one is a protein containing the DUF2789 domain, possibly linked to long-chain degradation, yet no function has been reported thus far. In the Dio vs. SA group, there are two genes with log_2_(FC) ≥ 5, namely the IucA/IucC family siderophore biosynthesis protein and the siderophore achromobactin biosynthesis protein AcsC, which are related to iron transport proteins and secondary metabolite biosynthesis. For genes with 1 ≤ log_2_(FC) < 5 in both groups, COG classification shows that they are mainly involved in lipid, inorganic ion, carbohydrate, and amino acid transport and metabolism (Figure 5). The transport and metabolism of amino acids and inorganic ions can modify the permeability of cell membranes to reduce the damage caused by the external environment to cells. Moreover, the transport and metabolism of carbohydrates help cells absorb alkanes and diesel for degradation. Compared to the control, genes related to cellular processes and signal transduction accounted for 21.95% in C16 group and 26.96% in Dio group. Upregulated gene expression here may be related to toxic substances during pollutant degradation. The cell membrane can effectively prevent their entry into cells and alleviate their toxic effects. When external toxic substances are produced, the upregulation of gene expression related to membrane formation represents a stress response of cells to changes in the external environment, minimizing the toxic impact on cells. In the Dio vs. C_16_ group, although diesel is more complex in composition than C_16_ and has a relatively shorter carbon chain, there are only 89 genes with upregulated expression (log_2_(FC) ≥ 1), which are mainly in various substance metabolic pathways like secondary metabolite biosynthesis and degradation.

### 2.5. Differential Expression of Alkane Hydrolases

**Figure 6:**
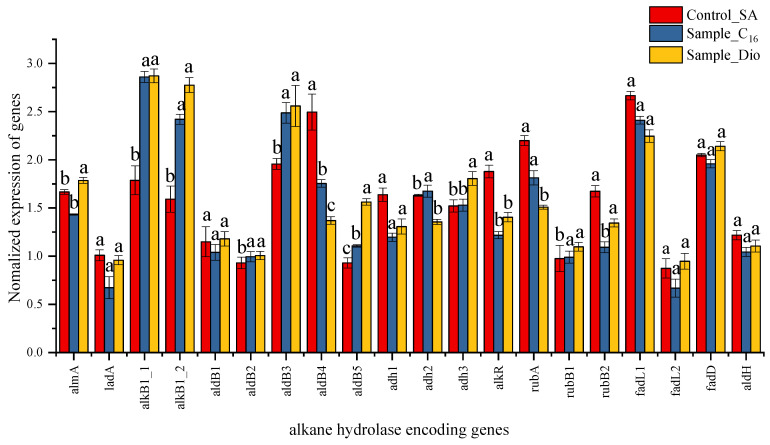
Expression of genes significantly regulated by C_16_, Dio, and SA in MSM. The normalized values were then used for logarithmic calculation using 2 as the base number. A value > 0 means gene upregulation, while a value < 0 means gene downregulation. Different letters indicate significant differences between pairs when both conditions of p-adjust < 0.05 and |log2FC| ≥ 1 are met.

Differential gene expression analysis (Figure 6) showed multiple alkane hydroxylases in strain KJ-1’s metabolism. Two alkane monooxygenase (AlkB1_1, AlkB1_2) encoding genes were significantly upregulated in the C_16_ vs. SA and Dio vs. SA groups. This is presumably due to the role that alkane 1-monooxygenase played in alkane hydroxylation [24,29]. When diesel and n-hexadecane acted as substrates, the expression levels of *alk*B1_1 and *alk*B1_2 had no significant difference, indicating broad substrate adaptability. The alkR gene upstream of the *alk*B1_1 showed downregulation with diesel and n-hexadecane compared to sodium acetate, suggesting it might downregulate the expression of *alk*B1_1. The long-chain alkane hydroxylase gene ladA did not exhibit significant expression differences in the C_16_ vs. SA and Dio vs. SA groups. In contrast, the almA was significantly upregulated in the Dio vs. SA group, yet no such significant upregulation was observed in the C_16_ vs. SA group. To verify the impact of the AlkB enzyme activity in *A. vivianii* KJ-1, AlkB1_1 was selected and a prokaryotic expression study of its encoding gene was carried out.

**Figure 7:**
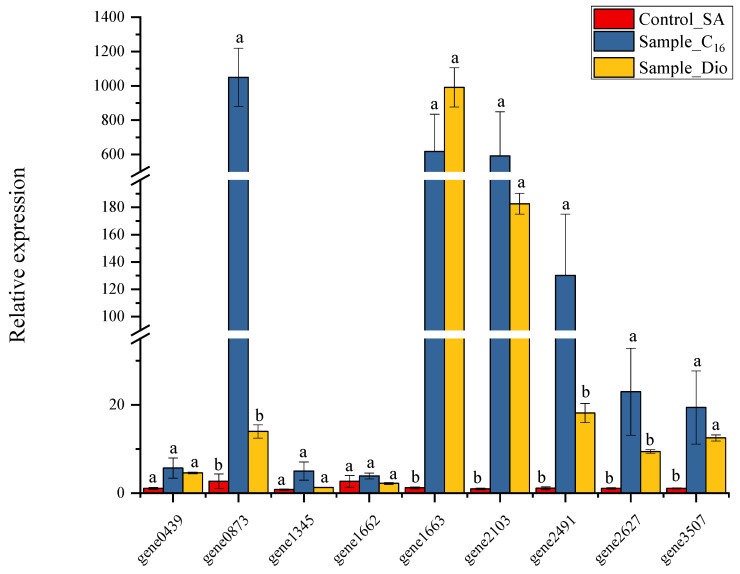
The relative mRNA levels of nine representative genes from *A. vivianii* KJ-1 under different treatments were determined by quantitative real-time polymerase chain reaction (qRT-PCR). The abundances of these genes were normalized with 16S rRNA as a reference. The genes whose expression are directly associated with n-hexadecane, diesel oil degradation, and transcriptional regulation were investigated. Different letters denote significant differences between pairs.

Analysis of other genes implicated in alkane degradation showed that not all relevant genes were upregulated in the transcriptome after alkane treatment (Figure 7). For example, the encoding genes associated with aldehyde dehydrogenase, namely aldB1 (gene0025) and aldH (gene1022), had little expression change under different substrates. Another aldehyde dehydrogenase-related encoding gene, ald4 (gene2740), was downregulated in hydrocarbon-treated (C16 and Dio) transcripts compared to those with sodium acetate (SA). Nevertheless, aldB2 (gene1003), aldB3(gene1345), and aldB5 (gene3595) were significantly upregulated. The proteins encoded by adh1 (gene1553), adh2 (gene1585), and adh3 (gene2920) belong to the alcohol dehydrogenase family. adh1 had minor expression differences among the three substrates, while adh2 and adh3 were significantly upregulated in n-hexadecane and diesel treated transcripts.

Other genes were involved in alkane degradation, such as rubredoxin (rubA) and rubredoxin reductase (rubB), which fulfill a critical function in the electron transfer process and contribute to the proper operation of alkane monooxygenases [29]. In the Dio vs. SA group, rubA and rubB1 showed differential expression with a negative correlation. FadL and FadD, responsible for the transportation of long-chain fatty acids, had similar expression levels in the transcriptomes treated with hexadecane and diesel.

**Figure 8:**
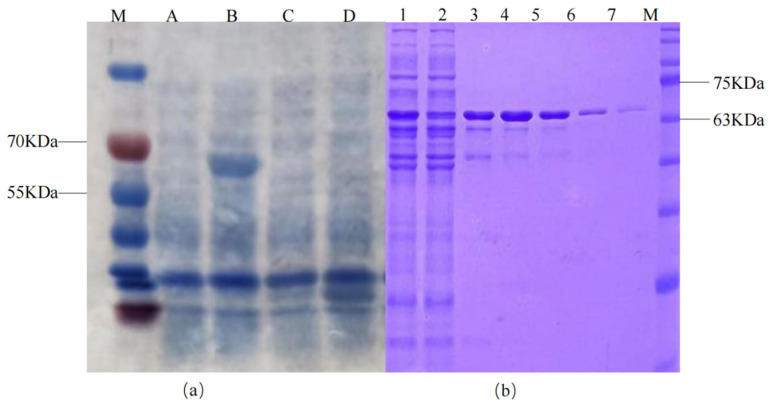
Expression and protein purification of recombinant plasmid induced by IPTG. (**a**): Expression of recombinant plasmid induced by IPTG; (**b**): Purification of recombinant protein. M: Protein marker; A and B: *E. coli* BL21(DE3): pSUMO1-*alk*B1_1 uninduced and induced expression; C and D: *E. coli* BL21(DE3): pSUMO1 uninduced and induced expression; 1: Pre-column protein 2: Flow-through after column 3–7: Elution fractions.

To explore the response relationship of the upregulated, differentially expressed genes (DEGs) under different treatments, quantitative real-time polymerase chain reaction (qRT-PCR) analysis was conducted on several genes (Figure 8). These included two alkane monooxygenase-encoding genes, an NAD(P)/FAD-dependent oxidoreductase-encoding gene, an aldehyde dehydrogenase family protein-encoding gene, an FAD-dependent oxidoreductase, an AMP-binding protein, an AraC family transcriptional regulator, and a putative protein-related gene. Results showed that the relative expression levels of the two alkane monooxygenase-encoding genes (gene1663 and gene2103) increased significantly with diesel and C_16_ as substrates. As a transcriptional regulator, *alkR* (gene1662) had no significant expression differences among these three substrates. Three alkane degradation-related genes, namely gene2911, gene2627, and gene3507, also had elevated expression, especially in the C_16_ group. Gene2491, related to the long-chain fatty acid transport protein, had a relatively high expression level in C_16_. In addition, gene0873, an unknown protein encoding gene, had a relatively high expression level in the C_16_ group and deserved further study in the next step.

### 2.6. Expression of Alkane Monooxygenase Gene

Primers were designed based on the alkane monooxygenase gene sequence. Subsequently, *alk*B1_1 (gene1663) was amplified. The prokaryotic expression vector pSUMO1-*alk*B1_1 and the recombinant strain *E. coli* BL21(DE3): pSUMO1-*alk*B1_1 were ultimately obtained. The protein was purified via the features of pSUMO1 (Figure 8). Figure 8 show that the IPTG-induced *E. coli* BL21(DE3): pSUMO1-*alk*B1_1 has a band at 64 kDa, which precisely aligns with the anticipated size of the alkane 1-monooxygenase protein. This indicates that target protein AlkB1 is efficiently expressed in the pSUMO1 vector and that alkane 1-monooxygenase is an inducible enzyme.

### 2.7. Enzyme Activity Assay

**Figure 9:**
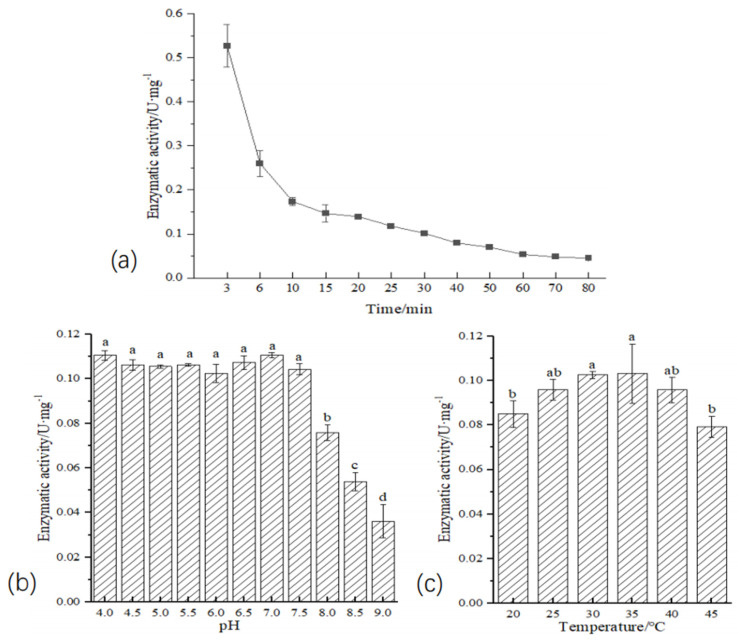
Enzymatic activity of recombinant *E. coli* BL21(DE3): pSUMO1-*alk*B1_1 (**a**): Enzymatic activity at different times; (**b**): The effect of pH on enzyme activity; (**c**): The effect of temperature on enzyme activity. Different lowercase letters above the bar chart represent significant differences among the treatments. The values are the means ± standard deviation (SD) of three replicates. The bar chart represents the standard deviation (n = 3; *p* ≤ 0.05).

The enzymatic activity of protein AlkB1_1 was measured over various time intervals (as delineated in Figure 9a). In the first 30 min, it dropped sharply, then the decline slowed. After 50 min, it stabilized. Therefore, 30 min was set as an experimental parameter to study the effect of temperature and pH. When pH was <7.5, the activity of the recombinant protein manifested no statistically discernible variance with pH values (one-way ANOVA F_6,14_ = 76.966, *p* > 0.05). However, when pH was ≥7.5, the enzyme activity of the recombinant protein started to decline significantly as the pH values increased (one-way ANOVA, F_3,8_ = 76.966, *p* < 0.001) (Figure 9b). As for temperature Figure 9c, the enzyme activity increased between 30 and 40 °C, attaining its peak at 35 °C. Temperatures above 40 °C or below 30 °C caused a reduction in enzyme activity (*p* < 0.05). However, the activity stayed above 0.07, showing that the recombinant protein AlkB1_1 can tolerate temperatures from 20 °C to 45 °C.

**Figure 10:**
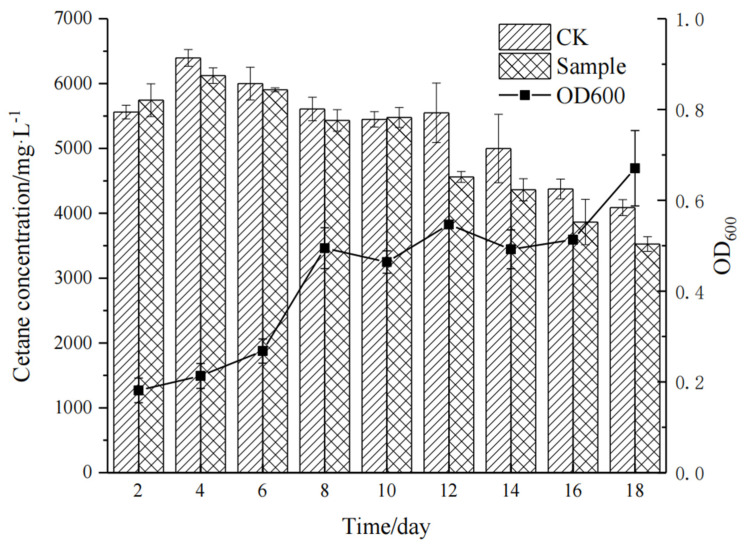
Degradation of n-hexadecane by recombinant strain *E. coli* BL21(DE3): pSUMO1-*alk*B1_1.

To determine if the recombinant protein could catalyze alkanes, the strain *E. coli* BL21(DE3): pSUMO1-*alk*B1_1 was incubated in inorganic salt medium with hexadecane as the sole carbon source. Samples were taken at set intervals to assess bacterial growth and quantify the residual hexadecane concentration (Figure 10). The recombinant strain grew rapidly from 6 to 8 days. In the first 10 days, there was no significant change. After 12 days, the residual hexadecane concentration dropped quickly, with a 17.82% degradation rate. By 18 days, it stabilized at around 13.8%.

## 3. Discussion

*Acinetobacter vivianii* KJ-1, a newly reported *Acinetobacter* strain capable of degrading petroleum hydrocarbons, shows a good degradation effect on diesel [11]. During the process of degradation of diesel with strain KJ-1, the short-chain alkanes C10–C12 in diesel gradually evaporated, and after treatment with KJ-1, their concentrations initially declined and then increased on the sixth day. This increase was hypothesized to result from the degradation of long-chain alkanes, which is consistent with the reduced levels of long-chain alkanes (C14-C26) in the experimental group compared to the control. The presence of numerous petroleum hydrocarbon-degrading enzymes in KJ-1, especially AlkB, suggests its crucial role in alkane degradation [26,28]. 

The genomic comparison among the four *Acinetobacter* strains revealed both similarities and differences. The relatively small fluctuation ranges in genome size and the number of protein-coding genes indicated a certain degree of genetic conservation with the genus. In the KEGG classification, through annotation with the KEGG database, it was found that the proportion of unique genes of the strain in the environmental information processes, especially in the two-component system as reported [11], is relatively high. Strain KJ-1, as isolated from it, is speculated to have a wide range of environmental adaptability. During the analysis of the genomic sequence, it was found that this bacterium has two *alk*B genes. Based on the gene sequence alignment [11], their protein sequences were also compared. Petroleum hydrocarbons, with their toxicity and degradation resistance, present a substantial threat to the environment [28]. The discovery of two genes encoding alkane 1-monooxygenase in *A. vivianii* KJ-1 is significant [11]. The consistent presence of six transmembrane helical regions in all eight AlkB proteins not only validates previous findings but also implies a conserved structural basis for their function as alkane monooxygenases [27,28].

This study on *A. vivianii* KJ-1’s DEGs under various substrates reveals insights into its metabolic responses. Comparing diesel and n-hexadecane to sodium acetate identified many DEGs, indicating that organic hydrocarbons impact alkane-related gene regulation [30]. The low number of co-up/downregulated DEGs (244/349) when C16/diesel is compared to sodium acetate emphasizes distinct transcriptional responses to different carbon sources, in line with previous research on bacteria’s diverse metabolic strategies [31]. The comparison between diesel and n-hexadecane (Dio vs C16) revealed a complex transcriptional landscape for diesel. The large number of differentially expressed features (DEFs) with a preponderance of downregulated genes (572 downregulated vs 92 upregulated) in the *A. vivianii* KJ-1 transcriptome suggests that diesel, with its more intricate composition, elicits a more complex transcriptional response. Similar findings have been reported in other studies where bacteria exhibited differential gene expression patterns in response to complex hydrocarbon mixtures [32]. *A. vivianii* KJ-1 preferentially uses short-chain alkanes in diesel medium, downregulating long-chain alkane genes, likely a strategic carbon-source-metabolizing adaptation. Cluster analysis validates distinct transcriptional patterns on C16 and diesel, reflecting metabolic requirements and regulatory mechanisms [33].

Identifying significantly differentially expressed genes (DEGs) reveals key insights into how the studied organism responds to different substrates [34]. In the C16 vs SA group, genes with high log₂(FC), like those encoding hypothetical proteins and the DUF2789-domain protein (linked to long-chain alkane degradation but uncharacterized), are of interest. Uncharacterized proteins can be important in specialized metabolic pathways, especially hydrocarbon degradation. DEGs in the Dio vs. SA group, such as IucA/IucC and AcsC, are related to iron transport and secondary metabolite biosynthesis. Iron is crucial for bacteria, and siderophores (produced by these genes) play a role in iron scavenging under iron-limited conditions [35]. This process may enhance hydrocarbon-degrading bacteria’s metabolism as many relevant enzymes are iron-containing. COG classification for genes with 1 ≤ log₂(FC) < 5 shows the significance of amino acid, inorganic ion, and carbohydrate transport and metabolism in cell adaptation and hydrocarbon degradation [36]. For instance, carbohydrate-mediated hydrocarbon uptake can boost degradation efficiency [37].

Differential gene expression in strain KJ-1 provides insights into its alkane metabolic pathways. The significant upregulation of AlkB1_1 and AlkB1_2 in the C16 vs SA and Dio vs SA groups is consistent with previous findings that alkane 1-monooxygenases play a crucial role in alkane hydroxylation [24,38]. Their similar expression with diesel and n-hexadecane shows broad substrate adaptability, beneficial in hydrocarbon-rich environments [24]. The *alk*R gene upstream of *alk*B1_1 was downregulated with diesel and n-hexadecane, suggesting it might negatively regulate *alk*B1_1, like in other hydrocarbon-degrading bacteria [39]. ladA showed no differential expression, while almA was upregulated only in the Dio vs SA group, highlighting the complexity of KJ-1’s alkane metabolic network. Studies [39,40,41] have shown that AlmA and LadA can oxidize long-chain alkanes (>C32) among n-alkanes. Wang [40] found that almA was upregulated not only by long-chain alkanes but also by branched-chain alkanes. Thus, it’s hypothesized that the upregulation of almA during diesel treatment results from the branched-chain alkanes in diesel components. 

The differential expression of genes involved in alkane degradation reveals the complexity of the organism’s metabolic mechanisms. AldB1 and AldH showed no upregulation during alkane treatment, suggesting their substrate-specificity or regulation by unactivated factors [42]. Ald4 was downregulated in hydrocarbon-treated samples, potentially due to negative feedback or a shift in metabolic priorities, as seen in other microbes when carbon sources change. In contrast, AldB2, AldB3, and AldB5 were upregulated, indicating their role in aldehyde processing during alkane degradation. The different expression of AldB 1-5, despite belonging to the same family, likely stems from their distinct aldehyde-chain-length specificities, like other hydrocarbon-metabolism enzyme families [43]. For alcohol dehydrogenase genes, Adh1 had minimal expression changes, suggesting a constitutive role, perhaps in long-chain alcohol oxidation. Adh2 and Adh3 were upregulated in n-hexadecane-and diesel-treated samples, respectively, implying specialization in medium-and short-chain alcohol oxidation, as reported in other hydrocarbon-degrading microbes [44]. Identifying genes like rubA and rubB, which are involved in the electron-transfer for alkane monooxygenases, is essential. Their differential and negatively-correlated expression in the Dio vs SA group points to a complex regulatory system. This balance likely fine-tunes the electron transfer, compensating for changes in the expression of one gene with the other. fadL and fadD expression in C16- and Dio-treated samples reveals the strain’s initial preference for short-chain alkanes, which is valuable for bioremediation in petroleum-contaminated sites as it aids in optimizing the process by targeting more degradable hydrocarbons. 

A prokaryotic expression study of AlkB1_1 was carried out to measure its activity and clarify its role, as achieved in prior studies [45]. Moreover, this study characterized AlkB1_1’s enzymatic properties and its role in alkane oxidation. However, further research is needed to fully understand its catalytic mechanisms and explore its potential applications, such as in bioremediating oil-contaminated sites.

## 4. Materials and Methods

### 4.1. Strains, Plasmids and Growth Conditions

The bacteria strains and plasmids utilized in this study are enumerated in Table 2. *Acinetobacter vivianii* KJ-1 (CGMCC No.20664) was isolated from the oil-contaminated soil in the proximity of a gas station and was taxonomically identified by means of physiological and biochemical characteristics in conjunction with 16S rRNA analysis [11]. *A. vivianii* KJ-1 and *E. coli* strains were cultivated on Luria–Bertani (LB) medium (comprising 10 g tryptone, 5 g yeast extract, and 10 g NaCl per liter) at 30 °C and 37 °C, respectively.

The diesel oil used in this study was obtained from a gas station of Shandong Kexin Oil Products Co., Ltd., in Jinan, China. The cetane index was 56, and the density was 827.9 g/cm^3^ (20 °C). The total pollutant content and the total sulfur content were found to be 4.5 mg/kg and 4.0 mg/kg, respectively. Sodium acetate, anhydrous sodium sulfate, n-hexane, n-hexadecane, and other chemicals and solvents were sourced from Shanghai Sinopharm Chemical Reagent Co., Ltd. (Shanghai, China) and Tianjin Guangfu Technology Development Co., Ltd. (Tianjin, China), all of which conformed to the highest grade of analytical reagents.

*A. vivianii* KJ-1 was inoculated into LB medium and incubated at 30 °C with continuous agitation at 150 rpm until an OD_600_ of 0.8 was attained. The cultures were then centrifuged, washed thrice, and resuspended in sterile water. To gauge the removal efficiency of strain KJ-1 for various alkanes in diesel oil, the cell concentration was adjusted to an OD_600_ of 0.6. The adjusted cell suspension was used to inoculate (2% volume ratio) 100 mL flasks of minimal salt medium (MSM) supplemented with diesel oil as the sole carbonaceous substrate. The MSM consisted of 1 g NH_4_NO_3_, 1 g NaCl, 1.5 g K_2_HPO_4_, 0.5 g KH_2_PO_4_, and 0.2 g MgSO_4_⋅7H_2_O per liter. The inoculated cultures were placed in a shaking incubator and shaken at 160 rpm. Samples were taken at different time intervals to determine the alkane concentrations in the diesel.

### 4.2. Comparative Genomic Analysis Based on the Whole Genome

The complete genome sequence of *A. vivianii* KJ-1 has been submitted and archived in GenBank under the accession number CP085083, with the associated BioProject ID *PRJNA768638* and the BioSample ID *SAMN22059268*. For comparative genome analysis, genome sequences of other typical petroleum hydrocarbon-degrading bacteria were retrieved from the NCBI database. Specifically, accession numbers GCA_002055515.1, GCA_000196795.1, and GCA_014250455.1 correspond to *A. calcoaceticus* CA16, *A. oleivorans* DR1, and *A. venetianus* Tust-DM21, respectively. All subsequent analyses were performed using the freely accessible online platform of the Majorbio Cloud Platform (www.majorbio.com accessed on 13 September 2020.) provided by Shanghai Majorbio Bio-pharm Technology Co., Ltd. (Shanghai, China) Genomic collinearity between strain KJ-1 and other *Acinetobacter* species was examined via the MUMmer3.23 software. Genomic synteny was then analyzed based on the alignment results. Comparative genomic analysis encompassed genomic synteny, core gene identification, specific gene identification, structural variation annotation, and genome visualization. The pan-genome and core gene functions were analyzed through PGAP v1.2.1, and the functional classification of core genes was grounded on the COG database. A Venn diagram was constructed to depict the relationships among the samples.

### 4.3. Transcriptome Sample Preparation and Analysis

The preparation of transcriptome samples is pivotal for deciphering the transcriptional responses of strains under diverse conditions. Analyzing strain transcriptomes upon exposure to different substrates is essential for a deeper understanding of the gene expression regulation mechanisms employed by these strains in varying environmental contexts. The cell density was adjusted to an OD_600_ of 0.6 and then used to inoculate (at a 5% volume ratio) 100 mL flasks containing minimal salt medium (MSM). Three different substrates were utilized as sole carbon sources: 0.3% (*v*/*v*) n-hexadecane (termed Sample_C_16_), 0.5% (*v*/*v*) diesel oil (Sample_Dio), and 0.5% (*m*/*v*) sodium acetate (Control_Y), with the sodium acetate culture serving as the control. All cultures were incubated at 150 rpm and 30 °C.

Total RNA was isolated from cells cultured under disparate conditions with TRIzol^®^ Reagent (produced by Invitrogen, Waltham, MA, USA) in accordance with the manufacturer’s instructions. Cells were harvested at a low temperature (4 °C, 12,000 rpm for 2 min) upon reaching the logarithmic growth phase. Specifically, this occurred at 3 days for the sodium acetate culture, 3 days for the diesel oil culture, and 4 days for the C_16_ culture. Transcriptome sequencing was implemented via RNA-seq technology. Differential expression analysis was performed to detect genes with a substantial change in expression levels. All experiments were repeated four times to guarantee the reliability and reproducibility of the results. Additionally, the 16S rRNA gene along with fifteen differentially expressed genes with known functions were selected for real-time quantitative reverse transcription PCR (qRT-PCR) analysis. The primer sequences employed for qRT-PCR are shown in Table S1.

### 4.4. Expression of the Alkane Monooxygenase Gene alkB1_1

**Figure 11:**
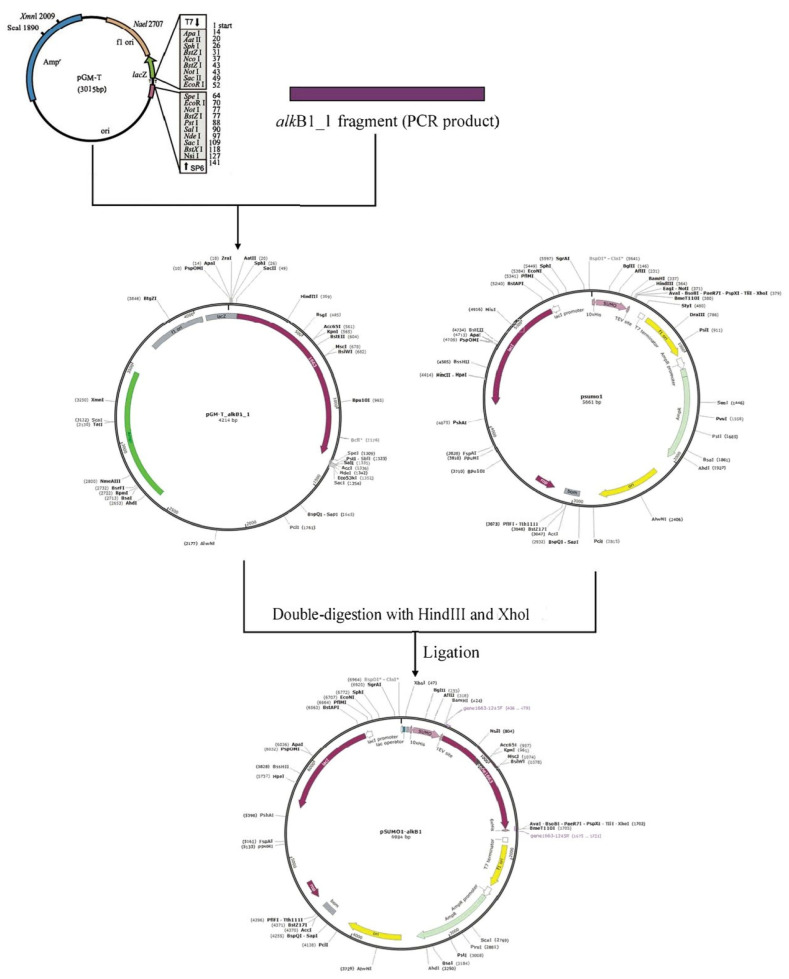
Flow chart of pSUMO1-*alk*B1_1 expression vector construction. Ligate the PCR fragment to the pGM-T to get pGM-T-*alk*B1_1. Digest pGM-T-*alk*B1_1 and pSUMO1 with HindIII and XhoI, then ligate the digested products to obtain recombinant plasmid pSUMO1-*alk*B1_1. “*” means BspDI and ClaI are isoschizomers.

The alkane monooxygenase gene *alk*B1_1 was subcloned into prokaryotic expression vector pSUMO1, procured from Jinan ABIOTECH biotechnology Co., Ltd. (Jinan, China), in China. The construction process of the expression vector is detailed in Figure 11. Subsequently, the plasmid pSUMO1-*alk*B1_1 was transfected into *E. coli* BL21(DE3) via the heat shock method for prokaryotic expression and subsequent activity analysis.

The primers employed for cloning the *alk*B1_1 gene are as follows.

- *alk*B1_1-F: 5′-CGAAGCTTCACCATGAATGCGCCAGTAAATGTAGAGC-3′
- *alk*B1_1-R: 5′-CGGAGCTCTGCAGTTGCTACTTGTTCTG-3′

### 4.5. Enzyme Activity Assay and Its Degradation of n-Hexadecane

NADH presents a characteristic absorption peak at 340 nm, whereas NAD^+^ does not absorb at this specific wavelength. During alkane utilization by AlkB, NADH is consumed, yielding NAD^+^ and thereby leading to a decrease in absorbance [46]. Spectrophotometry can be utilized to measure enzyme activity by meticulously monitoring the changes in absorbance as the reaction proceeds. This method is advantageous for determining the rate of enzyme activity decline and identifying the time point at which the enzyme activity stabilizes, which is essential for subsequent studies. The absorbance of NADH is measured under identical conditions in both the presence and absence of the *alk*B1_1 enzyme solution to determine the enzyme activity at various temperatures (from 20 °C to 45 °C) and pH levels (from 4.0 to 9.0). The enzyme activity assay system consists of the following components: 3 mL of Tris-HCl (with a concentration of 50 mM and a pH of 7.5), 1.5 μmol/mL of NADH, 10 mmol/mL of C_16_, and 1 mL of the enzyme solution. One unit of enzyme activity (U) is defined as the amount of enzyme that can consume 1 μmol of NADH per minute.

$$Enzyme\;Activity=\frac{10^{3}\times ∆A_{340}{\times V}_{T}\times D}{{6.22\times 10}^{3}\times T\times V_{E}\times L}$$

In the formula, Δ*A*_340_ represents the absorbance variation at 340 nm within a specific time interval, *V_T_* represents the total reaction volume, *D* corresponds to the dilution factor of enzyme solution used, and *V_E_* represents the volume of the enzyme solution employed. The value 6.22 × 10^3^ is the molar extinction coefficient of NADH at 340 nm. *T* represents the reaction time, and *L* represents the path length of the cuvette. 

To evaluate the degradation capacity of the recombinant strain for alkanes, hexadecane was employed as the sole carbon source with a concentration set at 0.3% (*v*/*v*). The optical density at 600 nm (OD_600_) values of the culture broth were monitored over time to determine the hexadecane degradation rate. These measurements were performed to confirm the hydroxylation activity of the recombinant protein on alkanes.

## 5. Conclusions

In this study, we used comparative genomics, transcriptome analysis, and prokaryotic expression of the alkB1_1 gene to explore how *A. vivianii* KJ-1 degrades petroleum hydrocarbons. Strain KJ-1 was highly efficient at degrading various alkanes in diesel oil, reducing the content of long-chain alkanes (C14–C26).

Comparative genomics showed similarities and differences between KJ-1 and other related strains. There was significant gene overlap, and a core genome of 2203 genes was identified. KJ-1’s genome had a relatively high proportion of uncharacterized genes.

Transcriptome analysis revealed that different substrates induced unique gene expression in KJ-1. When comparing C16 and diesel oil groups to the sodium acetate control, 1009 and 1275 differentially expressed genes (DEGs) were precisely identified. Upregulated DEGs were mainly related to the transport and metabolism of lipids, inorganic ions, carbohydrates, and amino acids, as well as cellular processes and signal transduction. Notably, alkB1_1 and alkB1_2, two alkane monooxygenase genes, were highly upregulated during C16 and diesel oil treatments, indicating their key role in alkane hydroxylation and broad substrate adaptability.

Prokaryotic expression and enzyme activity assays of the alkB1_1 gene showed that the recombinant protein AlkB1_1 was strongly expressed in the pSUMO1 vector and had inducible enzyme activity. The enzyme activity peaked at around pH 7.0 and between 30 and 40 °C, reaching its maximum at 35 °C. The recombinant strain was able to degrade n-hexadecane, further proving alkB1_1’s role in alkane oxidation.

Overall, this study provides valuable insights into the molecular mechanisms of petroleum hydrocarbon degradation by *A. vivianii* KJ-1. It also lays a solid theoretical foundation for improving bioremediation strategies for petroleum-contaminated environments.

## Figures and Tables

**Table 1:** Genome features of *Acinetobacter* spp. that degrade the petroleum hydrocarbon.

Sample Name	*A. calcoaceticus* CA16	*A. oleivorans* DR1	*A. venetianus* Tust-DM21	*A. vivianii* KJ-1
Genome Size (Mb)	4.12	4.15	4.12	3.93
Chromosome No.	1	1	1	1
Plasmid No.	1	0	3	0
G + C (%)	38.72	38.73	41.37	41.50
CDS	3879	3874	3846	3648
rRNA	18	18	18	18
tRNA	65	71	75	74
House-keeping gene No.	31	31	31	31
Genbank Number	GCA_002055515.1	GCA_000196795.1	GCA_014250455.1	GCA_020673335.1
Degradation substrate	n-alkane (where n = 12–18)	diesel oil	crude petroleum	diesel oil
Degradation rate	82–92%	60–80%	77.63–97.34%	>60%

**Table 2:** Strains and plasmids required for the experiment.

Strains or Plasmids	Characteristics	Source
Strains		
*E. coli* Top10	Suitable for efficient DNA cloning and plasmid amplification, ensuring stable inheritance of high copy plasmids	Lab Store
*E. coli* BL21(DE3)	Used to efficient expression of genes cloned into an expression vector containing a bacteriophage T7 promoter	TianGen-China
*A. vivianii* KJ-1	Wild-type, with diesel degradation capability	Lab Store
*E. coli* TO-*alk*B1_1	Amp^r^, TOP10 containing pGM-T-*alk*B1	This study
*E. coli* BL-*alk*B1_1	Amp^r^, BL21(DE3) containing pSUMO1-*alk*B1	This study
*E. coli* BL-C1	Amp^r^, BL21(DE3) containing pSUMO1	This study
plasmids		
pGM-T vector	Amp^r^, contains T7 and SP6 RNA polymerase promoters, and the polyclonal site region is located in β- Galactosidase α within the peptide coding region	TianGen-China
pGM-T-*alk*B1_1	Amp^r^, pGM-T vector containing gene *alk*B1_1	This study
pSUMO1 vector	Amp^r^, contains His-sumo tag	Abiotech-Jinan
pSUMO1-*alk*B1_1	Amp^r^, pSUMO1 vector containing gene *alk*B1_1	This study

## Data Availability

The data presented in this study are deposited in the National Center for Biotechnology Information (NCBI) repository, accession number GCA_020673335.1.

## Supplementary Materials

The following supporting information can be downloaded at: https://www.mdpi.com/article/10.3390/ijms26094083/s1.
