# Supplementary material for: Comparative Genomics, Transcriptome, and Prokaryotic Expression Analysis of alkB1_1 in Acinetobacter vivianii KJ-1: Revealing the Mechanism of Petroleum Hydrocarbon Degradation
Source: Int J Mol Sci. 2025 Apr 25;26(9):4083. doi: 10.3390/ijms26094083 (PMC12071677; doi:10.3390/ijms26094083)
Supplement: Supplementary file 1 [file ijms-26-04083-s001.zip › Table S1.pdf]

Table S1 Primers used for qRT-PCR.

Gene ID	Description	Primer name	Sequence (5'-3')
**gene0439**	NAD(P)/FAD-dependent oxidoreductase	gene0439-F	GGTGCAGGGATCTCAGGA
		gene0439-R	GAATAAATCCCAAGTCCC
**gene0837**	hypothetical protein	gene0873-F	GATCCAAGCGAGGCAGGT
		gene0873-R	ATCTGGATTGAGGCTGCC
**gene1345**	aldehyde dehydrogenase family protein	gene1345-F	TTCCCTGGAATTTCCCT
		gene1345-R	TGCAACCAGTAATATGCC
**gene1662**	AraC family transcriptional regulator	gene1662-F	GTCACGCTCCAAGTTTGC
		gene1662-R	GGCAATCTGTTGTATCGA
**gene1663**	alkane 1-monooxygenase	gene1663-F	ATTGAACACTACGGCCTA
		gene1663-R	AGCGTGATGATCTGAATGGCG
**gene2103**	alkane 1-monooxygenase	gene2103-F	GAACATTATGGTTTAAAGCGT
		gene2103-R	GTGATGATCAGAGTGGCG
**gene2491**	Transporter (Long-chain fatty acid transport protein)	gene2491-F	ATTGCATTGAAAGCGGCA
		gene2491-R	GTCGTTTTTGCGGCTTCG
**gene2627**	FAD-dependent oxidoreductase	gene2627-F	GTGAATGGTACTTTGCTTCCA
		gene2627-R	GACTGGCATGGCAGGGTA
**gene3507**	AMP-binding protein	gene3507-F	GGGTATTGGAACCGTCCA
		gene3507-R	GATCATGTCTTTCTTACGATC
**16S rRNA**		16S-F	GCCTAATACTGACGCTGAG
		16S-R	CCAGGCGGTCTACTTATCG
