# Supplementary material for: Comparative Genomics, Transcriptome, and Prokaryotic Expression Analysis of alkB1_1 in Acinetobacter vivianii KJ-1: Revealing the Mechanism of Petroleum Hydrocarbon Degradation
Source: Int J Mol Sci. 2025 Apr 25;26(9):4083. doi: 10.3390/ijms26094083 (PMC12071677; doi:10.3390/ijms26094083)
Supplement: Supplementary file 1 [file ijms-26-04083-s001.zip › Table S2.pdf]

Table S2 Data obtained from RNA-Seq.

Sample	Total raw reads	Total clean reads	Total mapping ratio
CK_1	8156566	8020158	97.05%
CK_2	8392368	8293958	93.61%
CK_3	8104174	8021962	98.26%
C16_1	8525490	7691520	90.63%
C16_2	8549922	7906056	93.63%
C16_3	8577614	7544822	95.25%
Dio_1	15604468	14419842	78.11%
Dio_2	7474722	7084306	80.97%
Dio_3	8065022	7675652	88.46%
